# Mineralization of Dentinal Lesions with Different Concentrations of Fluoride

**DOI:** 10.1155/2024/3476050

**Published:** 2024-03-20

**Authors:** Ayah A. Al-Asmar

**Affiliations:** Department of Restorative Dentistry, School of Dentistry, University of Jordan, Amman, Jordan

## Abstract

**Objectives:**

The present study aimed to investigate the relationship between fluoride concentration and mineral distribution within the dentinal lesion body.

**Methods:**

Remineralization of artificial deep dentinal lesions with various levels of fluoride was studied using a scanning electron microscope, microhardness tests, and polarized light microscope. Human molars were exposed to demineralization at pH 5.0 for 2 weeks. Then, they were divided into different groups for remineralization with different fluoride concentrations (0.1–10.0 ppm) for 1, 2, 3, and 4 weeks.

**Results:**

The results indicated a proportional relationship between fluoride concentration and dentinal lesion remineralization from 0.1 to 10.0 ppm. In the present study, the formation of a well-remineralized surface layer inhibited remineralization at the lesion front. On the other hand, the lesion front remineralization was found to be independent of fluoride concentration.

**Conclusion:**

Our results stated that for effective remineralization of dentinal lesions to the innermost part, fluoride levels from 1.0 to 5.0 ppm have the highest efficiency.

## 1. Background

In the last few decades, enamel remineralization has been a major subject of numerous studies [1–11]. The role of different fluoride concentrations in calcium phosphate–containing remineralizing solutions [12–16], toothpastes, varnishes, gels and dentifrices [17–22], glass ionomer cements [23–25], bonding agents [26], composites [27, 28], chewing gums [29, 30], and slow-release devices [31–33] in remineralizing incipient and advanced natural as well as artificial enamel lesions [3, 9, 34–36] is well-documented in the literature.

The thermodynamic driving forces and kinetic factors involved in enamel lesion formation have been intensively investigated and analyzed in situ and in vitro [37–50]. Moreover, remineralizing such lesions with various concentrations and forms of fluoride (sodium fluoride, stannous fluoride, monofluorophosphate sodium, acidulated phosphate fluoride, amine fluoride, silver fluoride, and silicate fluoride) to enhance remineralization has also been extensively studied [10, 51–58].

Relatively few studies have tested the remineralization phenomenon in dentin together with the effect of fluoride on remineralizing dentinal lesions. Although remineralizing dentin with fluoride-containing remineralizing solutions follows the same general physicochemical principles of enamel biomineralization, such a process is more complicated in dentin than in enamel due to the compositional and ultrastructural differences between both tissues. Dentin is composed of 20 wt% organic matrix while in enamel it is about only 1 wt%. Ninety percent of the organic phase in dentin is made up of collagen (mainly type I) while the remaining 10% are of noncollagenous components. In enamel, proteins form the major portion of the small inorganic phase. Moreover, the presence of dentin tubules, their orientation, numbers, and diameters influence dentin permeability and affect the diffusion process. Not only the volume but also the composition of the inorganic phase is different in dentin and enamel, 70 wt% in dentin and 96 wt% in enamel. The small dimensions of dentin crystallites, the proportions of carbonate and magnesium ions incorporated in the hydroxyapatite lattice, and their crystallinity and composition with dentin porosity complicate the remineralizing process even more. In addition to these differences, the dentin of mesenchymal origin is a biologically active tissue that forms one complex with the pulp through their histological, structural, and chemical interactions, unlike the ectodermal acellular enamel which is a biologically inert tissue.

The role of fluoride in dentin remineralization is of a particular interest for arresting root dentinal lesions [59–61] and repairing deep dentinal lesions under dental restorations [62–64]. Deep dentinal lesions are prone to remineralization under certain conditions which favour crystal growth on partially demineralized dentin [65–69]. Moreover, fluoride integration in minimal invasive dental therapies and biomimetic materials has been widely investigated recently [70–74]. Thus, fluoride application in several forms onto dentinal lesions is of clinical implication and importance in modern dentistry [75–77].

It is generally accepted that dentin is capable of remineralization but the distribution of mineral ions in the presence of fluoride within the lesion body and the depth at which the lesion can still be remineralized are not well clarified at present. The purpose of this study was to determine the level of fluoride that could enhance the remineralization of a dentinal lesion.

The role of fluoride in remineralizing the lesion surface, body, and front is to be studied together with the possible influence of the dense surface mineralized layer on remineralizing the lesion body and/or front. In this paper, we aimed to test the hypothesis that fluoride is capable of remineralizing the dentinal lesion front, and thus it is efficient in decreasing the lesion depth.

## 2. Methods

### 2.1. Sample Preparation

Seventy-five extracted human third molars were obtained from an oral surgeon's private clinic and used within 5 months of extraction. After extraction, teeth were immediately stored at room temperature in distilled water to which sodium azide was added to prevent bacterial growth. All teeth were clinically sound, and they were carefully observed for caries, abrasions, or any mechanical traumas. Teeth were cleaned with a toothbrush and sometimes with a scalpel to remove the periodontal ligament and intercrestal bone remnants and rinsed under running tap water. They were embedded individually in transparent cold-curing methylmethacrylate (Technovit 4004, Kulzer GmbH, Wehrheim, Germany). To expose deep-coronal dentin, the occlusal half of each tooth was cut using a slow-speed water-cooled diamond saw (Isomet, Buehler, Illinois, USA). Dentin exposed surfaces were then polished flat with waterproof silicon carbide abrasive paper (P500-grit) with Leco VP 100 (GmbH, Neuss, Germany). Subsequently, they were polished using wet polishing paper with a silicone paste of polycrystalline diamonds of size 9 *µ*m (DAP-7, Struers, Copenhagen, Denmark).

### 2.2. Lesion Formation

Dentin surfaces and the surrounding Technovit were coated with two coats of nail varnish (Keyte GmbH, München, Germany) to avoid the penetration of the solution into any marginal gaps that could exist between the tooth and the acrylate, while leaving two windows of exposed deep-coronal dentin per tooth. The adhesive paper was cut into 2 × 5 mm^2^ pieces and attached to the dentin surfaces before applying the nail varnish to standardize the windows' sizes. The samples were then kept in air for about half an hour to dry the nail varnish, and after the removal of the adhesive paper, they were immersed in the demineralizing solution (40 ml per sample). The demineralization solution contained 50 mM acetic acid, 2.2 mM CaCl_2_·2H_2_O, 2.2 mM KHPO_4_, 1 mM NaNa_3_, and 2 M KOH. No fluoride was added to the demineralizing solution. The pH was adjusted to 5.0 with drops of KOH and was measured throughout the demineralizing period (2 weeks) with gentle shaking (Müller Schüttler, München, Germany) at 37°C. The demineralizing solution was refreshed weekly to avoid changes of the solution's pH of more than half a pH unit.

### 2.3. Remineralization

After artificial lesion formation, the samples were washed with distilled water and divided into six groups (*n* = 12 per group). Each group was transferred to a flask containing 1 L of remineralizing solution composed of 20 mM HEPES, 1.5 mM CaCl_2_·2H_2_O, 0.9 mM KHPO_4_, 130 mM KCl, and 3.08 mM sodium azide with the pH adjusted to 7 with KOH. Different fluoride concentrations were used for each group 0, 0.1, 0.5, 1.0, 5.0, and 10.0 ppm as NaF. Again, remineralization was performed with shaking at 37.0°C. After the first week, the pH of the solutions was measured to be 7.2 for all groups, three samples were taken from each group and kept in Ringer solution until and during the processing period which always began on the same day, the solutions were refreshed, and the flasks were returned to the shaker once again. The same procedure was repeated every week for 4 weeks. The experimental groups are shown in Table 1.

### 2.4. Samples for Lesion Assessment

The teeth were cut perpendicularly to the two windows at the dentin surface with a thin diamond blade on a saw microtome (Leica SP 1600, GmbH, Nußloch, Germany) under tap water into thin (120 *µ*m) and thick (280 *µ*m) sections. Each section was then polished flat with wet silicon carbide abrasive paper (800-grit) to obtain a plano-parallel slice of 110 and 250 *µ*m thickness. Thin slices were used for imbibition in quinoline (Quinoline 22650, Fluka Chemie GmbH, Hamburg, Germany). They were then mounted for microscopic examination. Lesion depth was measured along a vertical line perpendicular to the tooth surface extending from a point at the lesion surface to a point at the nondemineralized surface throughout the lesion body to the innermost border of the lesion. The thick samples were divided into two groups. The first was taken for microhardness testing, and the second was prepared for morphological evaluation in a field emission scanning electron microscope (FE-SEM).

### 2.5. Analytical Tools

#### 2.5.1. Microscopy

Quinoline with polarized light (Axioskope 2, MAT, Carl Zeiss Jena GmbH, Göttingen, Germany) was used for the visual qualitative analysis of the lesions before and after remineralization. Digital images were taken with the image analysis software Axiovision (Rel. 4.4, SP2, Carl Zeiss Jena GmbH, Göttingen, Germany) for polarized light microscope (PLM).

#### 2.5.2. Microhardness

Testing the microhardness of the remineralized dentin was performed according to Marshall et al. [78] with a Vickers pyramid diamond indenter at 500 mN/mm^2^ and an automatic microhardness tester Fischerscope H100C (Helmut Fischer GmbH, Sindelfingen, Germany). Two lines were made per lesion in which each line was composed of 4–6 points which were spaced by 50–70 *µ*m. Each line extended vertically through the lesion from a point just beneath the lesion bottom up to the surface to determine cross surface microhardness (CSMH) throughout the lesion.

#### 2.5.3. FE-SEM

To obtain information on the morphology of the mineral depositions a FE-SEM was used. Samples were immersed in 50% alcohol for 20 min, then in 70%, 80%, and 90% alcohol each for20 min. Finally, they were kept overnight in 96% alcohol. Samples were immersed in hexamethyldisilazane for 10 min and air dried at room temperature according to Perdigao et al. [68]. Then, liquid nitrogen (−70°) was used for each sample for a few seconds to facilitate the fracture before using a scalpel to initiate a crack from the pulpal side. Each sample was then fixed on the SEM sample holder with carbon paste. Gold sputtering was done for 1 min; with 1.0 kV, 0.3 mbar, and 40 mA (Edwards Sputter Coater S15OB, Sussex, UK). Pictures were then made with a Leo FE-SEM (Leo DSM 982, Carl-Zeiss NTS GmbH, Oberkochen, Germany).

Throughout the whole experimental procedure, care was taken to avoid sample drying and dentin desiccation particularly after the lesion was formed except for the SEM samples where drying was mandatory.

## 3. Results

Lesion depth before and after remineralization with various fluoride concentrations after 1, 2, 3 and 4 weeks was measured by PLM and is shown in Table 2.

**Figure 1:**
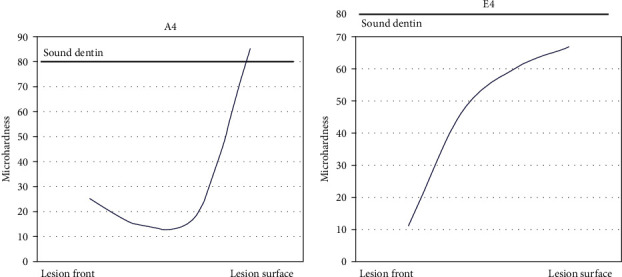
Microhardness representative profiles for Groups A4 (0.0 ppm fluoride, week 4) and E4 (5.0 ppm fluoride, week 4). Note the low microhardness values measured within the lesion body without fluoride in comparison with the high values when fluoride is added to the remineralizing solution (5.0 ppm).

Lesions that were remineralized without any fluoride additions to the remineralizing solution (Group A) showed no decrease in lesion depth either microscopically determined or with the microhardness profiles. Moreover, no changes were observed in the dentin hardness throughout the lesion even after 4 weeks (A4) of remineralization except for the surface layer where the Vickers indentations showed higher values. The surface values were equal to and sometimes even exceeded the values of sound dentin in the third (A3) and fourth week (A4; Figure 1).  Table 3 shows the mean values of the CSMH per group.

**Figure 2:**
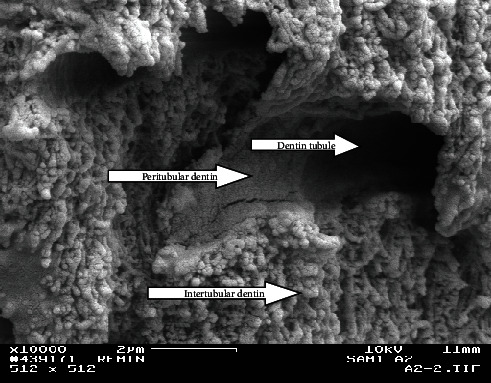
Fractured side from the upper most surface of the lesion from the control group at the second week (A2; ×10,000). Note the remineralized intertubular and peritubular dentin.

**Figure 3:**
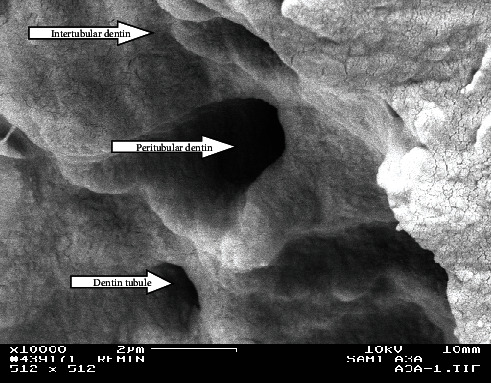
Fractured side from the upper most surface of the lesion from the control group at the third week (A3) (×10,000). Note the hypermineralized intertubular and peritubular dentin.

FE-SEM pictures of the surface of the control Group (A) showed well-mineralized intertubular dentin with some mineral precipitates at the surface. Peritubular dentin was also seen with tubules' diameters within the normal range (1.5–2.5 *µ*m), decreased or even occluded. Intertubular dentin at the fracture surface was more mineralized in Group A3 than in A2 as shown in Figures 2 and 3.

**Figure 4:**
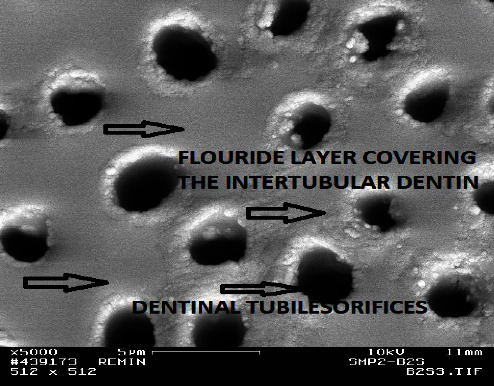
The surface of a remineralized lesion from Group B2 (0.1 ppm fluoride, 2 weeks). Note the well-remineralized intertubular and peritubular dentin (×5,000).

**Figure 5:**
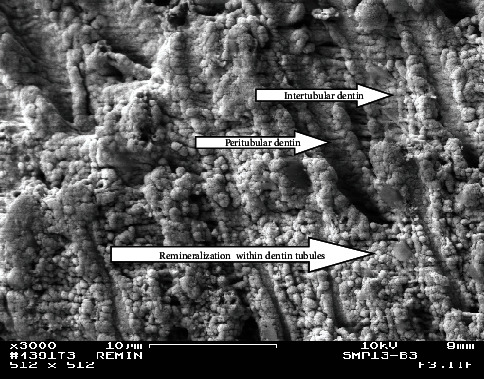
Fractured side of a lesion from Group B3 shows clearly the remineralized intertubular and peritubular dentin as well as remineralization within the dentin tubules (×3,000).

With the presence of fluoride ions in the remineralizing solution (0.1, 0.5, and 1.0 ppm), the distribution of minerals and the pattern of remineralization changed. After 1 week, there were no differences between the Groups B1, C1, and D1 and the control Group A1 in any of the used analytical tools except that the lesion depth was decreased by 40 *µ*m in the first three groups although the decrease was not constant in all samples and was not affected by differences in the fluoride concentrations. However, the SEM pictures of Groups B2, C2, and D2 showed much more surface mineralization with well-remineralized intertubular dentin and prominent thick peritubular dentin, and many of the dentin tubules were occluded as in Figure 4. Crystalline precipitates were also observed at the surface. The upper most surface layer of the fractured side appeared morphologically to be more mineralized than the remainder of the lesion body; although, the lesion body was also mineralized to the extent that borders of the dentin tubules within the lesion were not very distinguishable (Figure 5). Although the hardness tests did not demonstrate an increased hardness of the surface of these lesions, they showed an improved hardness in the lesion body in comparison with the control group. After the 4^th^ week, the hardness values were also increased at the surface but did not exceed the normal values.

**Figure 6:**
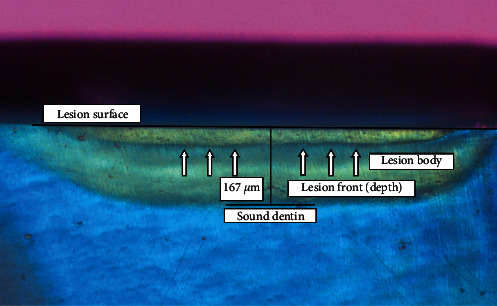
Remineralized dentinal lesion from Group C3 (0.5 ppm fluoride, 3 weeks) with polarized light microscope (×10). Note the remineralization band within the lesion (arrows). The method of lesion depth measuring is shown.

**Figure 7:**
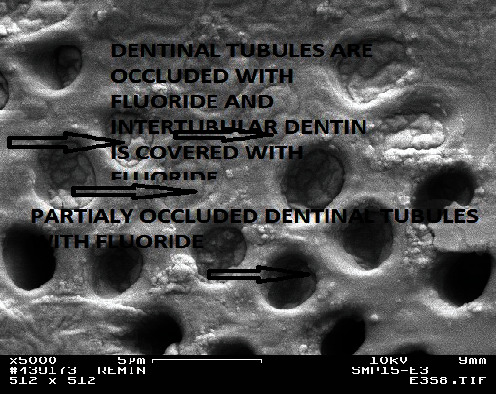
The occluded tubules at the surface of a sample from Group E3 (5.0 ppm fluoride, 3 weeks; ×5,000).

**Figure 8:**
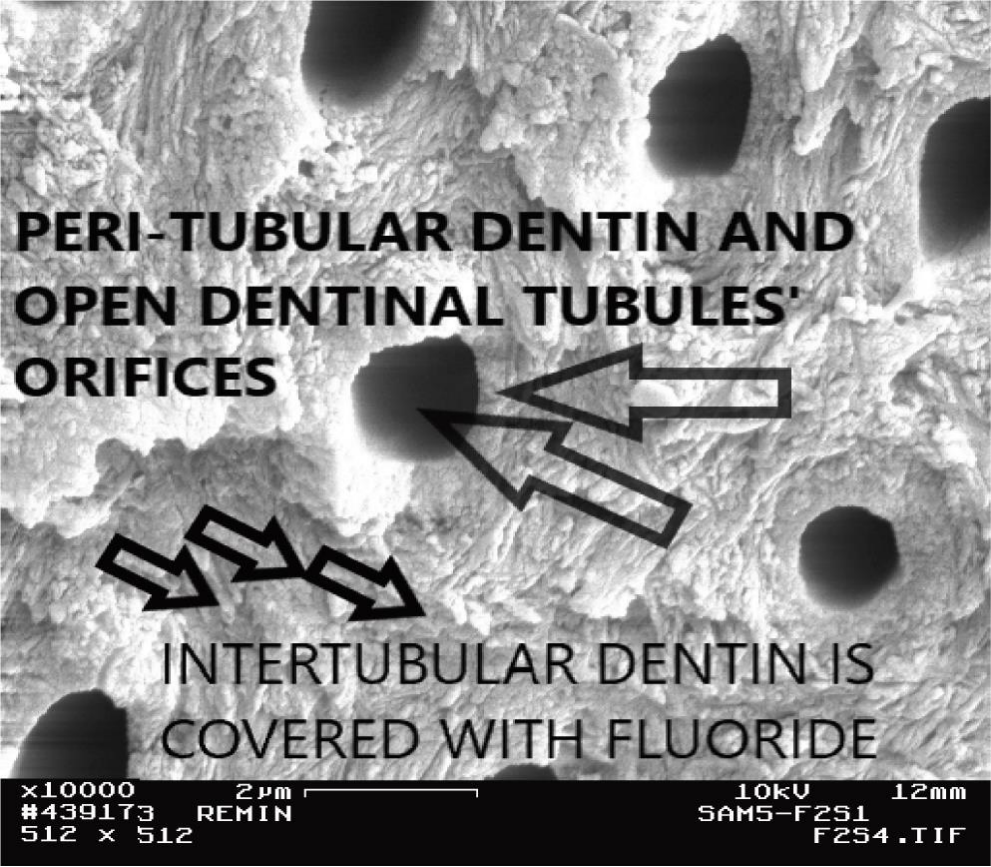
Hypermineralized intertubular dentin and thick peritubular dentin at the surface of a sample from Group F2 (10.0 ppm fluoride, 2 weeks; ×10,000).

**Figure 9:**
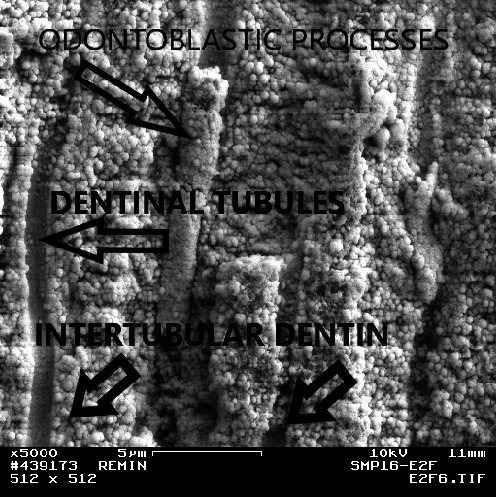
Remineralized precipitates (arrows) within the dentin tubules of the lesion body, the sample is from Group E2 (5.0 ppm fluoride, 2 weeks; ×5,000).

PLM showed banding near the surface and within lesion body in Groups B, C, and D (Figure 6). The banding seen with the PLM and the improved hardness measured by the Vickers indenter were strongly related to the fluoride concentration (D > C > B). Increasing the remineralization time also enhanced these effects (3^rd^ week > 2^nd^ week). After the 1^st^ week, there was no further decrease in lesion depth throughout the experimental period for all groups. Groups E and F had better hardness values from the 1^st^ week (E1 and F1). Moreover, an apparent surface layer appeared in the PLM, especially during the last 2 weeks (E3, E4, F3, and F4). Although the hardness measurement with the Vickers indenter did not show higher values at the surface in Groups E and F, the SEM pictures were full of precipitates that occluded the dentin tubules which were clearly surrounded with peritubular dentin and hypermineralized areas as in Figures 7 and 8. The hypermineralized areas were not continuous in Group E while they formed a dense hypermineralized layer in Group F. Mineral precipitates were also found at the fracture side within the lesion body in both groups (Figure 9).

## 4. Discussion

The current study investigated the effect of remineralization with various fluoride concentrations on the distribution of minerals throughout dentinal lesions. In the 90s, caries researchers such as ten Cate and Arends had focused on the effects of fluoride on dentin demineralization and remineralization in addition to their known studies of its effects on both phenomena in enamel. Cate et al. [15, 16] demonstrated that dentin has a much higher uptake capacity for fluoride than enamel, while Arends showed the ability of both bovine and human dentin to “over-remineralize” [27, 65]. However, in our study the remineralizing solution without fluoride addition did not contribute at all in remineralizing the lesion body together with its front. Moreover, a well-remineralized surface layer can still be formed even without fluoride and without observing a significant remineralization within the lesion body or decrease in lesion depth. These results were partially in agreement with Kawasaki et al. [75] who found that a surface layer was formed even without fluoride addition although this case showed a better overall remineralization than lesions which were remineralized in the presence of high fluoride levels (10 ppm). The difference in mineral distribution within the lesion in both studies can be due to the differences in the study's design and materials and methods so that a direct comparison between both studies cannot be made. First because his results were relative to other types of lesions in that study and second because the methods of evaluation used in the two experiments are not comparable because mineral deposition can occur within the lesion without contributing to its hardness [79]. In addition, differences in the structure and behavior between crown and root dentin due to differences in their development have been suggested by Goldberg and Smith [80]. Furthermore, the volume of the remineralizing solution per sample in the mentioned study was much smaller than ours which in return affects the remineralization rate.

In our experiment increasing the volume of demineralizing solution, demineralization duration, solution stirring, and refreshment probably resulted in an increased demineralization rate [81] and increased lesion depth with increased baseline mineral loss [82].

The increase in mineral loss increases the concentration gradient after putting the sample into a remineralization solution which in turn increases the initial remineralization rate [6, 55].

According to Fick's first and second laws, the flux of a material across a membrane is a function of both the concentration gradient (thermodynamic factor) as well as the diffusion coefficient (kinetic parameter). The rate of transport is faster when the concentration gradient is steeper [83]. The diffusion of mineral ions into and through the lesion is the rate limiting step for remineralization [56, 84]. Rapid precipitation of ions at the first reactive mineral surfaces leads to fast removal of the ions from the solution which retards any mineral deposition deep in the lesion [84]. In this case, fast precipitation at the surface of the lesion will prevent ions from reaching the innermost part of the lesion because of the sharp reduction of the thermodynamic force at the beginning and the blockage of pores at lesion surface later in the process [1, 35]. Therefore, ion precipitation can be also considered to be a rate limiting factor in the remineralization process where faster diffusion means faster precipitation at the surface which in return forms a dense mineralized surface layer which inhibits further diffusion. Thus, remineralization occurred at the top of the lesion (surface) first.

In the presence of fluoride, the overall remineralization pattern showed by FE-SEM, PLM, and through the microhardness measurements changed in terms of mineral distribution within the lesion, in which remineralization occurred at the bottom of the lesion (lesion front) first. Low levels of fluoride (0.1, 0.5, and 1.0 ppm) resulted in significant remineralization although this was not apparent in the 1^st^ week. Remineralization occurred at the lesion front as detected by decreased lesion depth under PLM. The higher hardness values which were evident throughout the lesion correlated well with the banding of the same lesions under PLM. No attempts were made to analyze the mineral bands shown in the microscope although some references suggest that fluoride is responsible for this lamination phenomenon in dentin [85, 86] and the total double refraction in water for enamel was correlated well with its mineral content [43]. Mineralized intertubular and peritubular dentin with the decreased in diameter or partially or totally occluded dentin tubules were clearly visible in FE-SEM pictures. According to the SEM and PLM pictures, the remineralization in the three Groups (B, C, and D) was enhanced with increasing the remineralization time (4^th^ week > 3^rd^ week > 2^nd^ week). In the present study, no attempts were made to compare directly between the three used methodologies since the information obtained from each quantifies a different physical property related to the tissue [43]. When fluoride was added to the remineralizing solution at higher concentrations (5.0 and 10.0 ppm) the SEM pictures revealed an obvious well-mineralized dentinal surface with dense precipitates accumulated in and on the intertubular and intratubular dentin as well as partially or totally occluded dentin tubules. These results are in agreement with other studies which demonstrated that fluoride ions presence catalyzes the precipitation of calcium and phosphate ions to penetrate the demineralized surface to remineralize the subsurface of dentinal lesions [87].

We tried to measure the thickness of the hypermineralized surface layer depending on its morphology for both Groups (E and F) from the fractured side. There were always differences in the measurements so that we could not estimate its thickness but we concluded that the hypermineralized surface layer in Groups E2 and E3 was not continuous because there were differences in its thickness within the same sample. Based on the SEM and PLM pictures and the microhardness values of Groups E2, E3, and E4, we hypothesize that the noncontinuous surface layer in these groups could not inhibit the diffusion process into the lesion or prevent lesion body remineralization. Our results are in agreement with Arends et al. [27] who found that the lesion front could be remineralized even after the formation of a hypermineralized surface layer using 5.0 ppm fluoride. A mineralized surface layer does not always prevent the deposition of minerals elsewhere in the lesion [68, 71]. In comparison, remineralization behavior in Groups F2, F3, and F4 were similar to the control groups in which a hypermineralized surface layer was formed without evident remineralization in the lesion body. This possible inhibitory effect of a hypermineralized fluoridated surface layer on the remineralization of the lesion front was stated by Kawasaki et al. [75].

No attempts were made to qualify the precipitated crystallites in and on the lesion surface. According to the literature, under conditions where fluoride levels are low and the pH is higher than 4.5, fluorohydroxyapatite10 or even fluoroapatite [88] have the highest probability to form.

Our results were very much similar to those found in literature regarding remineralization of the lesion front. In the absence of fluoride, remineralization did not appear to take place at the lesion front, and the lesion depth did not decrease [61, 69, 70]. Limited decrease in lesion depth after fluoride addition to the remineralizing solution was also previously documented [15, 27, 35, 56, 61, 63, 65, 84]. Various levels of fluoride (0.1–10.0 ppm) dramatically affected the surface mineralization. The surface remineralization was proportional to both fluoride concentration and duration of remineralization [27, 65].

We concluded from our results that incorporation of relatively small amounts of fluoride in the remineralizing process (0.5, 1.0, and 5.0 ppm) has the highest beneficial effect on dentinal lesion remineralization because such concentrations seem to be high enough to maintain a gradient at the lesion front, thus activating the thermodynamic driving force throughout the whole lesion. On the other hand, they are low enough to keep a constant diffusion rate to the innermost part of the lesion, thus controlling the kinetic of the precipitation process at least until the appearance of other inhibitory factors which spontaneously stop the process.

Such inhibitory factors could be:

1. The concentration gradient is not strong enough to maintain effective thermodynamics;
2. The rapid precipitation of ions at the first reactive surface areas of the dentinal crystallites which in turn blocks the lesion pores at the surface [1, 35, 70, 84];
3. The limited capacity of the dentinal front to remineralize, which is—most probably—due to the physical presence and chemical composition of the remaining organic phase where both properties can strongly restrict crystal growth [69, 76]. Hence, remineralization in this deepest area of the lesion is always limited and independent of fluoride concentrations.

Therefore, we suggest that neither number of available sites for remineralization alone [63] nor diffusion of ions solely [56] is completely responsible for controlling the remineralization phenomenon at dentinal lesion front.

Incorporating fluoride in precise concentration in biomimetic restorative materials to enhance deep dentinal lesions remineralization is of great impact on the capability of the material to remineralize the remaining demineralized dentin sufficiently. The authors suggest that clinical trials for such various fluoride concentrations embedded in dental materials and the consequent remineralization effect on dentinal lesions under restorations can be commenced shortly.

## 5. Conclusion

The present study indicates:

1. The influence of fluoride concentration in determining the rate as well as the pattern of mineral deposition in dentinal lesion.
2. The independence of lesion front remineralization from fluoride concentration which could be due to its limited capacity for remineralization.

## Figures and Tables

**Table 1:** After lesion formation, samples were divided into groups to be remineralized with various fluoride concentrations for different periods of time.

Group	Time			
	Week 1	Week 2	Week 3	Week 4
A (*F* = 0.0 ppm)	A1	A2	A3	A4
B (*F* = 0.1 ppm)	B1	B2	B3	B4
C (*F* = 0.5 ppm)	C1	C2	C3	C4
D (*F* = 1.0 ppm)	D1	D2	D3	D4
E (*F* = 5.0 ppm)	E1	E2	E3	E4
F (*F* = 10 ppm)	F1	F2	F3	F4

**Table 2:** Lesion depth before remineralization (BR) and after remineralization (AR) in each group (mean ± SD) as observed with the polarized light microscope.

	*F* level (ppm)	Time (days)	Lesion depth (*µ*m)
BR	≈0.0	14	210 ± 10

AR	0.0	7 14	195 ± 10 200 ± 15
	0.1	7 14	165 ± 20 168 ± 20
	0.5	7 14	170 ± 10 165 ± 15
	1.0	7 14	169 ± 15 167 ± 15
	5.0	7 14	165 ± 10 163 ± 20
	10.0	7 14	170 ± 30 169 ± 20

**Table 3:** The mean microhardness values measured with the Vickers indenter throughout the lesion per group.

Group	Time				
	Week 1	Week 2	Week 3	Week 4	CSMH values at the lesion surface at week 4
A	6.023	15.012	10.110	9.192	88.079 SD = 3.7
B	5.533	16.091	19.073	18.212	16.714 SD = 6.3
C	5.045	23.784	22.998	23.719	21.926 SD = 8.8
D	7.013	29.109	28.534	28.113	27.152 SD = 10.7
E	9.942	28.075	36.159	36.991	56.166 SD = 12.5
F	10.554	10.962	15.382	11.987	79.688 SD = 1.9

The average of the surface layer at week 4 for each group is given.

## Data Availability

The datasets used and/or analyzed during the current study are available from the corresponding author on reasonable request.

## Abbreviations

FE-SEM: Field emission scanning electron microscope
CSMH: Cross surface microhardness
PLM: Polarized light microscope.
